# Overview of protein posttranslational modifications in Arthropoda venoms

**DOI:** 10.1590/1678-9199-JVATITD-2021-0047

**Published:** 2022-04-15

**Authors:** Marcella Nunes de Melo-Braga, Raniele da Silva Moreira, João Henrique Diniz Brandão Gervásio, Liza Figueiredo Felicori

**Affiliations:** 1 Department of Biochemistry and Immunology, Institute of Biological Sciences, Federal University of Minas Gerais (UFMG), Belo Horizonte, MG, Brazil.

**Keywords:** Arthropod venom, Posttranslational modification, Glycosylation, Phosphorylation, PTM-venomics, Mass spectrometry-based proteomics, PTM-functional-venomics, UniProtKB/Swiss-Prot database

## Abstract

Accidents with venomous animals are a public health issue worldwide. Among the species involved in these accidents are scorpions, spiders, bees, wasps, and other members of the phylum Arthropoda. The knowledge of the function of proteins present in these venoms is important to guide diagnosis, therapeutics, besides being a source of a large variety of biotechnological active molecules. Although our understanding about the characteristics and function of arthropod venoms has been evolving in the last decades, a major aspect crucial for the function of these proteins remains poorly studied, the posttranslational modifications (PTMs). Comprehension of such modifications can contribute to better understanding the basis of envenomation, leading to improvements in the specificities of potential therapeutic toxins. Therefore, in this review, we bring to light protein/toxin PTMs in arthropod venoms by accessing the information present in the UniProtKB/Swiss-Prot database, including experimental and putative inferences. Then, we concentrate our discussion on the current knowledge on protein phosphorylation and glycosylation, highlighting the potential functionality of these modifications in arthropod venom. We also briefly describe general approaches to study “PTM-functional-venomics”, herein referred to the integration of PTM-venomics with a functional investigation of PTM impact on venom biology. Furthermore, we discuss the bottlenecks in toxinology studies covering PTM investigation. In conclusion, through the mining of PTMs in arthropod venoms, we observed a large gap in this field that limits our understanding on the biology of these venoms, affecting the diagnosis and therapeutics development. Hence, we encourage community efforts to draw attention to a better understanding of PTM in arthropod venom toxins.

## Background

Accidents with venomous animals are a public health problem worldwide, causing morbidity and, in some cases, death. The World Health Organization highlighted the impact caused by envenomation in 2017, with the reintegration of snake envenomation as a neglected tropical disease [1]. However, depending on the country, other venomous animals, such as arthropods, have a higher health impact. This is the case of Brazil, where more than 200 thousand accidents with arthropods were reported in 2019 [2]. The phylum Arthropoda (von Siebold, 1848) is the largest from the animal kingdom and includes members such as spiders, scorpions, bees, wasps, centipedes, and ants.

The symptoms and clinical manifestations caused by arthropod envenomation are induced by toxins and other venoms components. Animal venom is a complex and rich source of bioactive molecules, including peptides and proteins with a wide variety of functions, such as neurotoxins, serine proteases, hyaluronidases, metalloproteases, and insecticidal peptides [3,4]. The diversity of venom components is influenced by the species, animal age, gender, and the environment [5,6]. 

Although peptides/proteins from venom may be seen as a foe on envenomation, several components have remarkable therapeutic properties. Since the early 1980s, with the development of captopril, a hypertension drug derived from a viper snake peptide, venoms have been recognized as a “treasure chest”. This biotechnological potential is reinforced by 11 venom-derived drugs approved by FDA or related national agencies, and several molecules currently under preclinical phase and clinical trial [7]. This list includes arthropods molecules, among them, Apitox®, a whole bee venom used for osteoarthritis in South Korea since 2016. This drug also completed a clinical trial phase III study for knee osteoarthritis and it is in phase III for multiple sclerosis [7]. Thus, an extensive scientific effort is made to better access and characterize the molecular basis of envenomation. This new knowledge will expand the number of potential drug leads discovered and/or innovative envenomation treatments. 

The challenges involved in a complete characterization of venom biology are related to venom complexity. Advanced approaches such as the Omics, including the mass spectrometry-based proteomics, have contributed to this task and are often referred to as venomics [8,9]. Posttranslational modifications (PTMs) are an extremely relevant information that also contributes to the molecular complexity of arthropod venom proteins. PTMs influence the physicochemical properties of proteins, including their interaction with biological targets, and they are also a protective mechanism against proteolysis since they confer higher protein stability [10]. Despite the classical toxin PTMs such as disulfide bond and amidation, more than 300 known PTMs are rarely explored in arthropod venomics. Different PTMs can also be present in the same protein to modulate biological outcomes, resulting in significantly higher complexity. The study related to the functional implications of PTM mechanisms in envenomation, including their potential impact on venom toxicity, is still poorly understood in toxinology. Besides its physiological impact, the manipulation of toxin PTMs for therapeutic purpose is widely used to improve stability, solubility, affinity and to alter immune response [11].

Consequently, the question here is, what do we know about protein PTMs in arthropod venom so far? Therefore, in this review, we will focus on modified venom toxins/proteins using the gold standard UniProtKB/Swiss-Prot Protein Database [12,13] as it frequently contains manually curated entries. In addition, we will focus on the current knowledge on venom protein phosphorylation and glycosylation in these venomous animals. We also briefly describe analytical approaches in PTM-venomics and biological approaches that can be used to infer the role of PTMs in venom activities. The integration of such studies we refer to as “PTM-functional-venomics”. Finally, we will discuss the foreseen challenges associated with PTMs studies in toxinology.

## Posttranslational modifications of arthropod venom proteins included in the UniProtKB/Swiss-Prot database

The inclusion of known PTMs descriptions in protein databases is essential to retrieve such information and to have a broader view of venom biology. Among the databases with such information, the UniProt is the most comprehensive one [13], including the UniProtKB/Swiss-Prot Tox-Prot program, which integrates proteins and toxins from a wide variety of venomous organisms [12]. Within Expasy, a bioinformatics resource portal of the Swiss Institute of Bioinformatics (SIB), the free web platform named VenomZone (https://venomzone.expasy.org/) is available with a direct link to UniProtKB/Swiss-Prot. This website is an attractive and updated platform reporting 16 PTMs classification, among other annotations, from six animal taxa. Since we wanted to expand the PTM knowledge for the entire Phylum Arthropoda, we performed a customized search in UniProtKB/Swiss-Prot using the following search parameters: “taxonomy:arthropoda (annotation:(type:"tissue specificity" venom) OR locations:(location:nematocyst))”. As of 31^st^ of January 2021, we retrieved 3,066 entries of venom proteins/toxins. Next, we retrieved the entries containing PTM information by searching for “keyword:"PTM [KW-9991]" (taxonomy:arthropoda (annotation:(type:"tissue specificity" venom) OR locations:(location:nematocyst)))”. Strikingly, 90% of all reported arthropod venom proteins contain PTM information (2,759 out of 3,066). 

**Figure 1.**
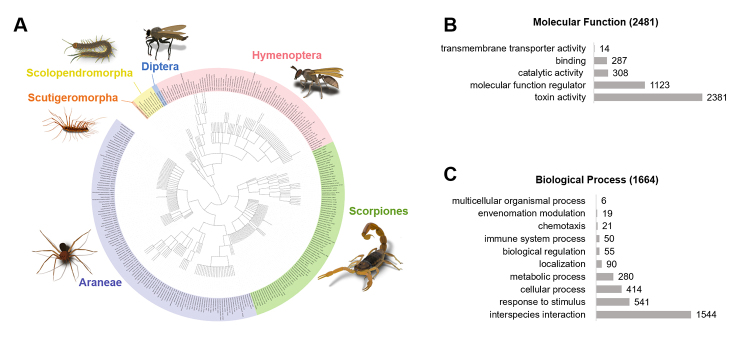
Arthropod venom proteins containing posttranslational modification (PTM) information in the keyword subsection of UniProtKB/Swiss-Prot Protein Database. **(A)** Phylogenetic tree of arthropods species, with PTMs information in the database (experimental plus putative), grouped by Order. Purple corresponds to Araneae, green to Scorpiones, pink to Hymenoptera, blue to Diptera, yellow to Scolopendromorpha and orange to Scutigeromorpha. **(B)** Gene ontology classification of arthropod venom proteins containing PTMs information based on their molecular function from 2481 entries and **(C)** biological process from 1664 entries. Phylogenetic figure was created in the interactive Tree of Life web server (http://itol.embl.de) [14].

This PTM dataset comprises 304 species from 47 families belonging to six orders (Araneae, Scorpiones, Hymenoptera, Scolopendromorpha, Diptera, and Scutigeromorpha) (Figure 1A and see Additional file 1). The proteins/toxins have a molecular weight range from 786 Da to 159.12 kDa, where 68.4% of them were below 10 kDa. Consistent with general venom biology, this dataset is enriched with toxin activity and molecular function regulators (Figure 1B). In addition, the dataset displays a remarkable diversity of biological processes, including response to stimulus, cellular and metabolic processes, localization, biological regulation, immune system, among others (Figure 1C). 

**Figure 2.**
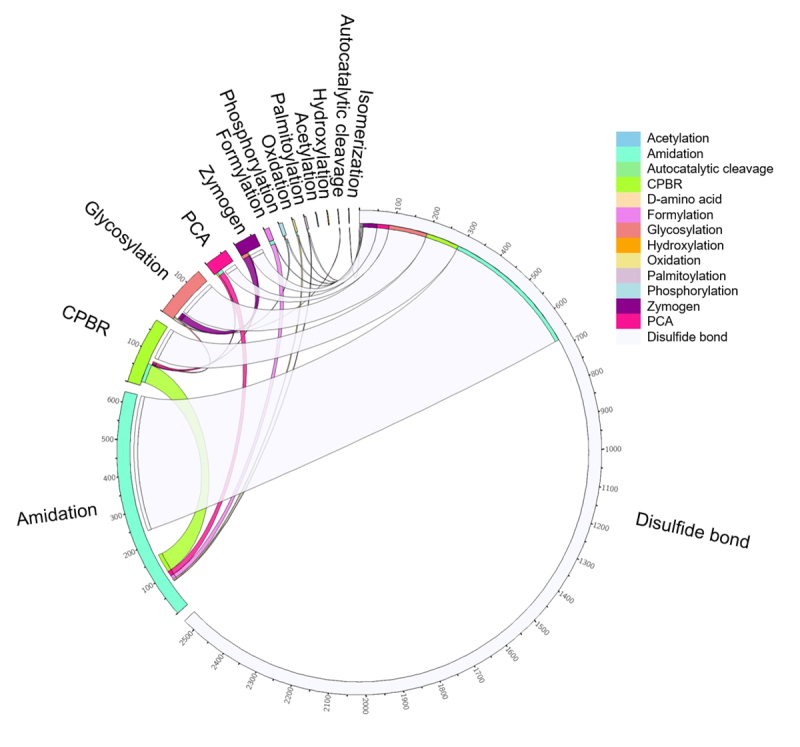
Protein posttranslational modifications (PTMs) in arthropod venom included in the UniProtKB/Swiss-Prot Protein Database. Circos table viewer representation of 14 PTM types identified in all the arthropod venom proteins from UniProtKB/Swiss-Prot Protein and their dual interaction when multiple PTMs were identified in one entry. The circle represents the entries with specific modification, taking into consideration different PTM combinations. This data set includes experimental and putative PTMs. The image was created in http://mkweb.bcgsc.ca/tableviewer/ [15]. PCA: pyrrolidone carboxylic acid; CPBR: cleavage on pair of basic residues.

The retrieved PTMs included 14 classifications: acetylation, amidation, autocatalytic cleavage (AC), cleavage on pair of basic residues (CPBR), disulfide bond, formylation, glycosylation, hydroxylation, isomerization (D-amino acid), oxidation, palmitoylation, pyrrolidone carboxylic acid (PCA), phosphorylation, and zymogen (pro-enzymes) (Figure 2 and Additional file 1). Hymenoptera was the Order containing proteins with more PTMs diversity, followed by Araneae and Scorpiones (see Additional file 1).

Disulfide bond and amidation were the most representative PTMs, which were found in 2,489 UniProtKB/Swiss-Prot entries (90.2%) and 610 entries (22.1%), respectively (Figure 2). Although 74.3% of proteins/toxins contain only one PTM in the UniProtKB/Swiss-Prot keyword field, multiple PTMs information was observed in 25.7% of the entries (Figure 2 and Additional file 1).

PTM annotations from UniProtKB/Swiss-Prot are based on distinct evidence [13]. Therefore, we classified the protein modification in this dataset as experimental or putative, investigating the information on the PTM/Processing subsection. We only included as experimental those with the modified site linked to a scientific publication or stated as a combined source with experimental and computational data. The putative PTM information is based on sequence similarity, sequence analysis, manual assertion inferred by curator, manual assertion based on curator opinion, or when the information appears exclusive in the PTM-keyword section. Only the CPBR and zymogen were not classified. 

Most of the PTM information from UniProtKB/Swiss-Prot entries mined in this work are putative (Table 1 and Additional file 1). Interestingly, the amidation was the better characterized one, followed by disulfide bond and PCA (Table 1). Although the putative information reinforces the potential presence of PTMs, there is a long journey to access them experimentally in venom proteins/toxins, as we will discuss later in this review. However, we can highlight some PTM functionality in arthropod venom biology from experimental evidence. Disulfide bond confers tridimensional conformation stability to support a range of biological functions [16,17]. Protein/toxin amidation increased affinity for voltage-gated ion channels as the sodium channels, leading to higher toxin potency and toxicity [18-20]. Also, amidation has a role in antimicrobial activity [21]. N-terminal acetylation was related to the inflammatory activity of wasp polybine peptides, revealing higher chemotaxis of PMNL cells and mast cell degranulation than the unmodified [22]. Isomerization with D-amino acid is a strategy to increase resistance to proteases and increase the potency of toxins, as reported for the spider omega-agatoxin-TK toxin, an inhibitor of P-type calcium channel [23,24]. A “spontaneously” methionine oxidation of tertiapin, a honey bee potassium channels inhibitor, blocks its interaction with GIRK1/4 and ROMK1 channels [25]. Palmitoylation plays a positive role in the spider PLTX-II toxin biological activity, in which the enzymatic removal of palmitoylation leads to decreased toxin potency [26]. Although we did not classify CBPR and zymogen, together with autocatalysis cleavage, they are classical proteolytic processes frequently observed in venoms toxins as a strategy to activate or maturate some proteins/toxins [27,28]. Proteolysis is often overlooked as PTM but is highly important in venom biology, being associated with molecular diversity in scorpion venom [29]. Cryptic peptides or cryptides are biologically active peptides that can be generated from larger peptides or proteins via proteolysis [30]. For example, Rocha-Resende et al. [31] reported that the scorpion neurotoxin Ts3 undergoes proteolysis to yield a vasoactive cryptic peptide in the venom of *Tityus serrulatus*.

We also identified phosphorylation and glycosylation in this dataset, including venom proteins/toxins with both modifications (Figure 2 and Additional file 1). While these modifications are the most frequently reported in UniProtKB/Swiss-Prot [32], they are underrepresented in arthropod venom. Notably, in snake venom, glycosylation modulates proteins/toxins activities [33] and it was suggested as a molecular signature candidate to venom phenotype [34]. Considering the importance of these modifications in other organisms and their feasible high-throughput study, we explored the current knowledge on their potential role in arthropod venom biology. In the next sections, we will describe these PTMs information included in the UniProtKB/Swiss-Prot and in some additional scientific publications related to the topic.

**Table 1:** Summarized data compiled from UniProtKB/Swiss-Prot of PTM information in arthropod venom proteins/toxins.

PTM Type	Total entries	Experimental	Putative
Disulfide bond	2489	263	2226
Amidation	610	359	251
Cleavage on pair of basic residues	166	nc	nc
Glycosylation	118	11	107
Pyrrolidone carboxylic acid	58	45	13
Zymogen	40	nc	nc
Formylation	12	2	10
Phosphorylation	6	4	2
Oxidation	5	4	1
Palmitate	3	2	1
Acetylation	2	2	0
Autocatalytic cleavage	1	0	1
D-amino acid	1	1	0
Hydroxylation	1	1	0

nc: not classified

## Phosphorylation of arthropod venom proteins

Protein phosphorylation is a widely studied PTM in humans, since this modification displays multiple roles in the regulation of cell cycle, protein synthesis, protein degradation, cell differentiation, proliferation, and apoptosis [35]. However, somehow this important PTM is still overlooked in the arthropod venom proteins and in toxinology. Thus, there are still open questions about whether phosphorylation plays a role in arthropod venom adaptive evolution. In this PTM, a phosphate group is added or removed from the side chain of specific amino acid residues (e.g., serine, threonine, and tyrosine) by regulation of kinases and phosphatases, respectively. 

Phosphatase activity in spider venoms has been known for a long time [36,37]. The presence of phosphatases is not exclusive to spiders, and they were also observed in scorpions [38,39], bees [40], wasps [41], among others. Kinase enzymes were already identified in the venom of spiders [42], wasps [41,43,44], honey bees [45], scorpions [46], and ants [47]. Arginine kinase was found in these studies, suggesting that Arg may also be phosphorylated in arthropods. However, several additional kinases were identified in *Polybia paulista* wasp venom, such as serine/threonine-protein kinases, tyrosine-protein kinase, and dual-specificity protein kinases [41]. The identification of these phosphorylation regulatory enzymes in venoms reinforces the potential of such modification in their peptides/proteins.

There are six phosphorylated peptides/proteins from arthropod venoms in UniProtKB/Swiss-Prot. Four of them were experimentally identified, in which three are from scorpions (hypotensin-1, hypotensin-2 and toxin To46) and one from wasp (venom allergen 5, Ag5) venoms. Two are putative by similarity, one from wasp (also an Ag5) and one from spider (acetylcholinesterase-1, AChE). All peptides/proteins were identified with a single phosphorylated site on serine in scorpion and spider peptides/proteins or tyrosine residues in wasp proteins. These proteins/toxins vary from peptides with ten amino acid residues (toxin To46) to proteins containing 559 amino acid residues (AChE). 

Verano-Braga et al. [29] identified the three mentioned phosphorylated scorpion toxins in the first high-throughput PTMome study of the *T. serrulatus* venom - herein called “PTM-venomics”. Hypotensin-1 and hypotensin-2 have dual activity as bradykinin (BK) potentiating peptides and agonists of B2 receptors [48,49]. However, it is still unknown if phosphorylation might directly or indirectly affect their hypotensive effects. Interesting to note, the C-terminal fragment (KPP) of hypotensins is essential to keep their dual activity, but phosphorylation occurs at Ser^6^ near to the N-terminal. Regarding the toxin To46, phosphorylated at Ser^8^ [29], there is no evidence or speculation of its potential target or function in the literature. Besides these toxins, the authors identified additional 128 phosphorylated peptides using a bottom-up venomics approach [29], using titanium dioxide (TiO2) enrichment. However, no annotation was associated with their sequence. 

Santos-Pinto et al. [50] performed a venomics of the wasp *Polybia paulista* leading to the characterization of different proteoforms of Ag5, including one of them phosphorylated. This study was based on 2DE gel-proteomics with TiO2 enrichment of selected protein spots to investigate phosphorylation and proteolysis with a diverse array of enzymes. This Ag5 cysteine-rich venom protein containing four disulfide bonds was also found with different modifications besides phosphorylation at Tyr^111^. The phosphorylated proteoform of mature Ag5 was suggested to occur in a relatively high abundance in nature (1:1 as modified *vs*. unmodified counterpart) [50]. This modified mature form was recognized by a pool of human specific-IgE. Although the exact role of Tyr^111^ phosphorylation in this protein is still unknown, allergen with multi phosphorylation was associated with decreased affinity to specific-IgE, attenuating allergic reactions [51]. The other Ag5 from *Vespa velutina* was identified in UniProtKB/Swiss-Prot as putative by sequence similarity with the previously mentioned one, but with phosphorylation at Tyr^107^. 

Besides the UniProtKB/Swiss-Prot identifications, other arthropod phosphorylated proteins/toxins are identified in the literature, such as melittin, icarapin and phospholipase A2 (PLA2) [40,52] that seem to have an important role in allergy. Phosphorylated melittin (Ser^18^) had lower toxicity compared to its native form, shown by lower mast cell degranulating activity, hemolytic activity, and reduced chemotaxis (leukocyte migration). However, no difference in the lytic effect was observed in mast cells. This result reinforces a potential modulation of allergy for arthropod phosphorylated toxins. Melittin was also identified phosphorylated at Thr^10^ and future investigation of this proteoform is necessary. PLA2 hydrolyze acyl esters from phospholipids, mediating several biological activities, including platelet aggregation, inflammation, hemolysis, necrosis, circulatory failure and others, besides the hypersensitivity reaction [53]. Phosphorylation of PLA2 was identified in bee venom [52]. In humans, this PTM is relevant for enzyme activity [54,55] by interfering in the PLA interaction with its inhibitor [56]. However, there are no studies on phosphorylation functionality in arthropod venom PLA2. 

From spider venoms, the acetylcholinesterase-1 (AChE) was reported in UniProtKB/Swiss-Prot with putative phosphorylation at Ser^223^ by sequence similarity, with no publication or homolog protein reference. This enzyme is responsible for the degradation of the neurotransmitter acetylcholine (Ach), playing a pivotal role in cholinergic transmission in the neuronal synapse and neuromuscular junctions. The *in vivo* and *in vitro* phosphorylation were reported for the human AChE, where its phosphorylation enhances Ach hydrolysis [57]. Since Ach is the primary excitatory neurotransmitter in insect central nervous system (CSN) [58], AChE phosphorylation may be important for predation, but further investigation is necessary. Notably, phosphorylation was also identified in the bee neurotoxin apamin [52], also not included in UniProtKB/Swiss-Prot. This venom toxin targets calcium-activated potassium channels, blocking their activity, which regulates excitability [59,60]. These results also reinforce a potential neuro-modulation by phosphorylation in arthropod venom proteins. 

Besides the natural occurrence of phosphorylation in venom peptides/proteins, phosphorylation can occur ex-situ and in cellulo. Ronjat et al. [61] showed for the first time that a cell-penetrating scorpion venom peptide (maurocalcin, MCa) undergoes phosphorylation in ex-situ by PKA kinase and in the HEK293 cytoplasm after penetration. MCa is a potent agonist of intracellular ryanodine receptor type 1 (RyR1), leading to calcium release. However, the MCa phosphorylation at Thr^26^ leads to the opposite effect, in which MCa becomes a RyR1 antagonist. This fascinating study opens a new perspective on venom peptides/proteins that undergo phosphorylation within host cells as drug leads. However, it is important to keep in mind that the biological effect depends on the studied system. For instance, MCaThr^26^-Phospho maintains the same MCa native biological activity in intact muscle fibers [62]. As an explanation, the authors suggested a phosphatase activity in fibers, which converts the phosphorylated MCa into its active proteoform [62]. The manipulation of phosphorylation was also reported in other toxinology research, as observed for the Kv1.3 channel synthetic inhibitors derived from the sea anemone ShK peptide [63]. The specificity toward Kv1.3 over Kv11.1 was 35-fold and 104.2-fold higher by D-phosphotyrosine or L-phosphotyrosine incorporation, respectively [63]. The improvement in the latter made possible its further application to treat psoriasis, an autoimmune disease, with promising results in the clinical trial [64]. 

In summary, there is evidence of the neuro-immune modulation role of phosphorylation in arthropod toxins, especially in allergenicity. However, the impact of phosphorylation on vasoactive peptides as hypotensin and other processes is still obscure. The manipulation of this PTM can also be explored to alter the activity, toxicity, and/or improve the specificity of potential therapeutic toxins. It is important to point out the lack of information regarding the presence and the role of phosphorylation in arthropods’ venoms. In addition, it is necessary to stress the need to confirm the putative PTMs and improve the functional characterization of several identified phosphorylated proteins/toxins.

## Glycosylation in arthropod venom proteins

The most complex and diverse PTM is protein glycosylation, in which carbohydrate units are covalently linked to specific amino acid residues in proteins. Although there are different glycosylation types, we will focus on N-glycosylation, O-glycosylation, and glycation, since they were identified in arthropod venom. The N-glycosylation occurs in the side chain of asparagine residues within the NXS/T/C consensus sequence, where X can be any amino acid residue except proline. The O-glycosylation occurs in the side chain of serine and threonine residues, while glycation, non-enzymatic glycosylation, occurs in the side chain of lysine residue or in the amino-terminal group [65,66]. These modifications alter the conformation, solubility, and stability of proteins, and they play a role in cell interaction, communication, molecular recognition, and cell signaling.

As observed for phosphorylation, several glycosylation modulatory enzymes were identified in arthropod venoms, such as alpha-glucosidase, alpha-mannosidase, beta-galactosidase, 3-beta glucuronosyltransferase, protein glycosyltransferase, and aldose 1-epimerase [40,41]. The presence of these enzymes in arthropod venom is an additional confirmation that their proteins/toxins potentially undergo glycosylation. 

**Figure 3.**
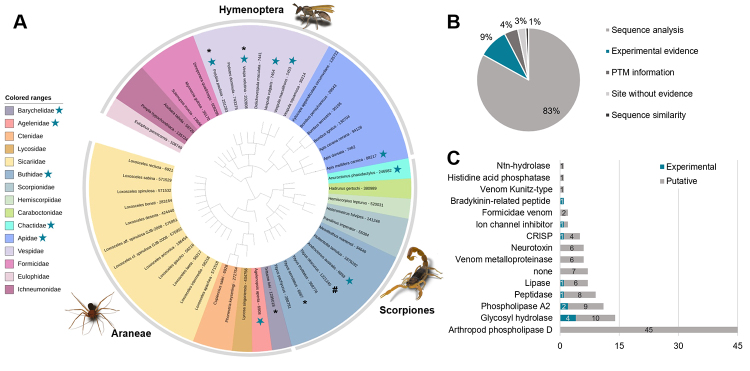
Arthropod venom proteins glycosylation and phosphorylation from UniProtKB/Swiss-Prot Protein Database. **(A)** Phylogenetic tree of arthropods species that contains phosphorylation and glycosylation information in the database. The species are grouped in colored range by Family taxonomy and the outer circle shows the Order they belong to. 🞴 Species containing proteins with both glycosylation and phosphorylation information. **#**Species with proteins presenting only phosphorylation information. (Family and species with experimental glycosylation and/or phosphorylation. **(B)** The database contains different evidence of 118 protein entries containing glycosylation information. **(C)** Distribution of 111 glycoproteins (experimental and putative) among different protein families provided by Uniprot based on the InterPro database [67].

There are currently 118 arthropod venom proteins/toxins with PTM-keyword glycosylation in UniProtKB/Swiss-Prot. Most of them are from spiders (with 63 entries from 17 species/strains), followed by hymenopters (41 entries from 21 species) and scorpions (14 entries from 11 species) (Figure 3A). However, most of the experimentally identified are from hymenopters (Figure 3A). Despite the identified number, only a few contain experimental evidence, representing 9% of this dataset (9 entries with N-linked, 1 entry with O-linked, and 1 entry with glycation), while 91% are putative based on different evidence (104 N-glycosylation, 1 glycation and 2 without any glycosylation type description) (Figure 3B). The putative information is divided into five categories, with the sequence analysis being the majority (83%) since N-glycosylation occurs in the NXS/T/C motif (Figure 3B). Indeed, putative glycosylation is the most identified PTM in Uniprot [32]. In five entries (4%), the putative glycosylation information appears in the PTM-keywords and in the posttranslational modification subsection but without reporting the glycosylation type and position (Figure 3B). Two of them were identified based on similarity and three with linked publication but without characterization. These entries are mainly fragments from larger proteins such as venom protease, hyaluronidase, U-myrmeciitoxin and aspartylglucosaminidase. 

The 118 proteins/toxins are distributed among 15 protein families (Figure 3C) and we will discuss the putative and experimental ones separated. Among the putative results, the arthropod phospholipases D are by far the most representative ones. Veiga et al. [68] showed the potential biological relevance of glycosylation in phospholipases D or SMase D. The enzymatic removal of N-glycosylation decreased the dermonecrotic activity in spider *Loxosceles intermedia* venom [68]. However, no identification of glycosylated proteins was performed in this study and, to the best of our knowledge, there is no experimental confirmation of glycosylation in SMase D. Therefore, the remaining question is whether the dermonecrotic activity diminished by the direct impact of deglycosylation of SMase D or the structural impact caused by deglycosylation or even by the effect of glycosylation from other proteins as hyaluronidases. 

Metalloproteinase family was also identified with putative N-glycosylation sites (Figure 3C). In the same work, Veiga et al. [68] observed a reduced gelatinolytic activity after *L. intermedia* venom deglycosylation, suggesting the presence of glycosylated metalloprotease. Although experimental confirmations are still necessary, these enzymes might undergo glycosylation by homology with metalloproteases from snake venom [33,69]. Remarkably, glycosylation in metalloprotease from snake venoms enhances its toxicity, since removing such modification leads to loss of hemorrhagic activity. In contrast, the proteolytic activity on fibrinogen and fibronectin was not altered [69]. This example clearly illustrates that different proteoforms might evolve to target distinct molecules. Besides the UniProtKB/Swiss-Prot data, a zinc metalloproteinase-disintegrin-like protein was stained for glycosylation in *P. paulista* wasp venom [43], confirming its presence in arthropod venom. However, better site and functional characterization are necessary.

From UniProtKB/Swiss-Prot, members from the protein families of phospholipase A2 (PLA2), lipase (e.g., PLA1), glycosyl hydrolase (e.g., hyaluronidases), CRISP (e.g., Ag5), ion channel inhibitor (toxin Aah6), peptidase (e.g., isomerase), and bradykinin-related peptide (Vespulakinin-1) were experimentally identified (Figure 3C). PLA1 and PLA2 were both identified with N-glycosylation. These enzymes are important components in arthropod venoms, as mentioned before. A scorpion PLA2 was identified with three N-glycosylated sites (Asn^43^, Asn^101^, Asn^153^) using lectin affinity chromatography [70]. Its carbohydrate composition was evaluated by mass spectrometry and included three hexoses, two N-acetyl-hexoses (GlcNAc), and two deoxyhexoses, but the presence of mannose was also suggested. The structures of 14 N-glycans were also identified from honeybee PLA2 using lectin affinity [71]. The oligosaccharides include paucimannose (Man3-GlcNAc2 or Man4-GlcNAc2) with fucose, and hybrid with two fucoses, additional GlcNAc linked to mannose and a terminal N-acetylgalactosamine (GalNAc) [71]. High mannose was also found in PLA1 from ant, showing a role in IgE cross-reactivity with honeybee venom [72]. The glycosylation of PLA1 and PLA2 was also characterized in wasps with a possible immunomodulatory role [41,73,74]. Although non-glycosylated PLA might preserve or even increase its activity, and also be recognized by IgE, glycosylation seems to be important for induction and maintenance of PLA sensitization [75]. A similar result was observed for PLA2 from bee venom, where protein structure, enzymatic activity and allergenic activity were independent of glycosylation. However, glycosylation seems to enhance allergenic activity by increasing IgE reactivity [76]. In humans, glycosylation of PLA was related to activity and secretion [77]. In general, the heterogeneity of glycosylated proteoforms might lead to distinct activities or affinities toward their targets. 

The hyaluronidase has a diffusing role by increasing the permeability of venom components from the sting local into the bloodstream and then target organs. Thus, hyaluronidase increases toxins toxicity upon arthropod envenomation (see review [78]). Anti-hyaluronidase was shown to reduce venom-induced lethality, suggesting the important role of this type of glycosylated protein in the envenomation process [79]. Hyaluronidases have several putative N-glycosylation sites, but this modification was characterized only in a few arthropods, including spiders [80], wasps [73,81,82], and honeybee [83]. Heterogenous glycoforms were also reported for hyaluronidases, including fucose presence [80,82,83]. The lack of glycosylation apparently does not affect IgE recognition either the activity of recombinant hyaluronidases, while influencing protein aggregation [78]. In addition, glycosylation was associated with optimum pH and thermal stability of wasp venom hyaluronidase activity. The recombinant enzyme without glycosylation exhibited highest activity at pH 2 with significant loss of activity in pH 3, while the native hyaluronidase showed activity from pH 2 to 10 with optimal activity at pH 6 [84]. Thus, it seems that glycosylation has a role in hyaluronidase toxin stability. 

Besides phosphorylation, glycation was also found in wasp allergen Ag5 at Lys^141^ by venomics, including HILIC enrichment [50]. The glycated Ag5 was estimated to be 3-fold more abundant compared to its non-modified counterpart [50]. The glycated Ag5 was also reactive to specific IgE, suggesting additional glycosylation immunomodulatory role in allergic reaction [50]. In addition, the glycation in Ag5 might be the reason behind the observed broad cross-reactivity against different Hymenoptera venom [41].

The sodium channel toxin Aah6 was the first glycosylated neurotoxin characterized from scorpion [85]. This toxin has heterogeneous N-glycosylation at Asn^9^, with a common glycan core structure of GlcNAc(α1-6Fuc)(α1-3Fuc)(β1-4GlcNAc). The authors observed structures from high mannose to short glycan fragments [85]. Moreover, potassium channel toxin alpha-KTx 21.1, known as Tityustoxin-15 (Ts15), is glycosylated at Asn^27^ and contains three disulfide bridges. Although Ts15 is included in UniProtKB/Swiss-Prot as putative glycosylation, this toxin was identified as glycosylated in the *Tityus serrulatus* PTM-venomics but with no glycan characterization [29]. As the name suggests, this toxin targets potassium channels blocking its ion current [86]. However, the function of glycosylation in these ion channel inhibitor toxins was not currently addressed to the best of our knowledge. Thus, further investigation is necessary to achieve glycosylation influence in toxin-ion channel affinity, stability, solubility, or proteolysis resistance. 

In the same *T. serrulatus* PTM-venomics, more 97 N-glycosylated peptides were identified by bottom-up venomics, but with no protein annotation. The glycopeptides were enriched by TiO2 (sialylated glycopeptides) and hydrophilic interaction liquid chromatography (HILIC) strategies. Interestingly, 69% of the identified glycopeptides were suggested to contain sialic acid since they were enriched using TiO2 [29], but no glycan structure information was reported in this study.

The Vespulakinin-1 from *Vespula maculifrons* wasp venom is O-glycosylated at Thr^3^ and Thr^4^ [87]. In rat *in vivo* assays, this bradykinin-related peptide could contract the uterus and ileum while promoting duodenum relaxation. This vasoactive peptide was at least 2-fold more potent than bradykinin (BK) to reduce blood pressure, but no significant difference was observed in the effect duration [87]. The double glycosylated analog of Vespulakinin-1 was also more active than BK and the non-glycosylated analog in mammalian smooth muscle and on insect CNS presynaptic block neurotransmission [88]. This result indicates the potential role of O-glycosylation in predation and in vasoactive peptides. 

Notably, glycosylation was also reported in PTMs regulatory enzymes as isomerases [89,90]. Also, Souza et al. [41] identified several phosphorylation and glycosylation modulatory enzymes (e.g., kinase, phosphatase, glycosidase, and glycosyltransferase) that undergo glycosylation in *P. paulista* wasp venom. The authors speculated on the importance of N-glycosylation in wasp venom, including improvement of protein/toxin stability, escape from the victim's immune system and from different inhibitors displayed in preys [41]. However, more investigations are still necessary to explain the role of glycosylation in arthropod venoms. The manipulation of glycosylation in toxins analogues with potential therapeutic applications is also an alternative strategy to improve proteolytic stability, peptide/toxin activity and selectivity toward tumor cells [91].

## PTM-functional-venomics approaches

To obtain new insights into biological systems, including venomous animals, improvement and development of technologies were necessary for high throughput studies. In this sense, mass spectrometry-based proteomics has become widely used for protein/PTM identification and characterization. In addition, the next-generation sequencing (NGS) applied nowadays in genome and transcriptome has expanded our knowledge on genes and transcripts reports and allowed straightforward proteomics application. Therefore, the combination of Omics methodologies has emerged as a powerful way for discovering unknown components in a large-scale manner to study venom biology [17,92-96]. 

**Figure 4.**
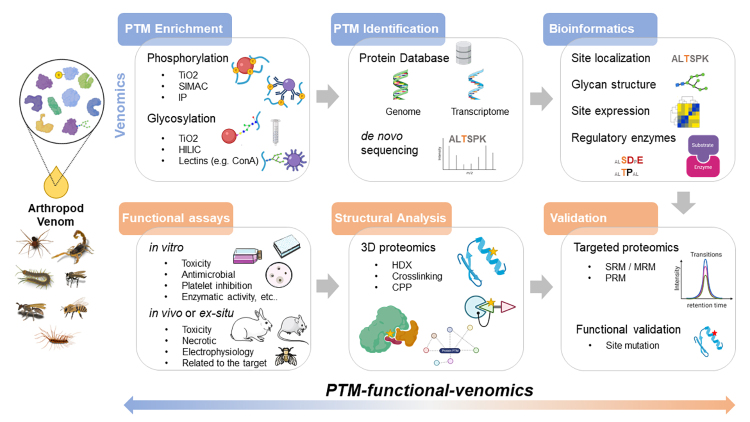
PTM-functional venomics. General strategies based on venomics studies on protein phosphorylation and glycosylation and the integration with functional assays and structural proteomics to investigate PTM biological/functional impact on venom activities. Target proteomics and site mutation can be performed to validate PTM assignment and PTM function, respectively. HDX: hydrogen/deuterium exchange; CPP: covalent protein painting; SRM: selected reaction monitoring; MRM: multiple reaction monitoring; PRM: parallel reaction monitoring. Figure created with elements from BioRender.com and Servier Medical Art.

In PTM-venomics, there are different approaches to explore venom biology. Protein separation can be gel-based or gel-free, and their identification is based on mass spectrometry analysis of peptides (bottom-up), proteins (top-down), or larger peptides (middle-down). A more comprehensive PTM characterization, including potential multiple PTMs, might be obtained by top-down venomics, allowing the identification of different proteoforms. Although recent advances in top-down application in toxinology were reported [97], this technique is still a challenge to be used as a routine in most laboratories. The reasons are difficulties in protein separation, protein fragmentation in mass spectrometry, among others. Therefore, we will discuss the bottom-up approach to study venom proteins and PTMs (Figure 4). In this approach, the proteins are cleavage with specific enzymes, like trypsin, but the use of orthogonal enzymes (e.g., Glu-C, Asp-N) should be considered to increase peptide variability and, thus, protein coverage.

PTM analyzes are challenging and the main reasons are the sub-stoichiometric abundances of proteins carrying PTMs compared to their unmodified counterparts and their highly dynamic nature. A way to overcome such difficulties is using efficient PTM enrichment strategies. Although it is still possible to identify modified proteoforms in venoms without enrichment steps [29,50], it is undeniable that enrichment methodologies enhance the identification of PTMs. In addition, the combination of enrichment and pre-fractionation methods allows the identification of several thousands of PTMs in recent days. Since PTMs have a high dynamic range, their complete coverage is still far from reality, and improvements are still necessary, including better sample preparation methods, even more sensitive and fast instruments [98,99]. So far, any enrichment strategy allows the unbiased selection of a specific PTM, and the combination of different approaches is complementary to achieve better coverage.

In accordance with this review scope, we will briefly mention some enrichment approaches to study phosphorylation and glycosylation. Several established strategies to enrich phosphopeptides can be applied to toxinology, including antibody-based methods (immunoprecipitation, IP), affinity chromatography (e.g., TiO2, SIMAC), chemical derivatization, and phosphopeptide precipitation (Figure 4) [100-102]. Among them, the TiO2 affinity was used to identify some of the arthropod phosphopeptides mentioned before [29,50]. This method can be combined with other enrichment strategies such as the immobilized metal affinity chromatography (IMAC) to perform sequential elution from IMAC (SIMAC), enhancing the identification of multi phosphorylated peptides [103]. Importantly, when studying phosphoproteome, it is necessary to add phosphatase inhibitors, together with protease inhibitors, at the beginning of venom preparation. 

The characterization of glycoproteins can be performed either by obtaining the glycan structure information or just by accessing the peptide sequence after enzymatic removal of the glycosylation. For instance, PNGase F can be used to remove N-glycosylations, which leads to the conversion of Asn into Asp with an increment of 0.9840 Da [104]. The study of the intact glycoproteins presents many analytical challenges. For example, we can highlight glycan heterogeneity, the poor ionization in electrospray (ESI), and large fragment ions generated by MS/MS [105]. Although glycosylation is a common modification found in nature, several enrichment strategies improve the sensitivity to study this modification. Lectin affinity chromatography, as concanavalin A, hydrazine affinity purification, hydrophilic interaction liquid chromatography (HILIC), and even the TiO2 chromatography are examples of glycoprotein/glycopeptide enrichment methods (Figure 4) [105-107]. Among them, concanavalin A, HILIC, and TiO2 were used to investigate glycosylation in arthropod venoms [29,41,50]. TiO2 has an additional affinity toward glycopeptides containing the negatively charged sialic acid (SA) [108,109], while HILIC allows the selection of neutral and sialylated ones. 

Besides the study of a single PTM type, the study of multiple PTMs is becoming routine in many groups for simultaneous PTMs interplay investigation. Although still a challenge, the combination of different approaches with sequential enrichments is often applied to address multiple-PTMs. For instance, phosphorylated peptides and sialylated glycopeptides can be simultaneously enriched using TiO2 [110]. This approach was also applied to study PTM in scorpion venom [29]. Furthermore, the TiO2 flow-through can be used to study the unmodified peptides for the total proteome approach.

After enrichment steps and high-resolution mass spectrometry analysis (LC-MS/MS), the following step is the bioinformatic analysis. There are several software for protein identification, such as MaxQuant, Proteome Discoverer, among others. The search can be performed against protein databases such as NCBI and Uniprot or customized databases based on genes and transcripts from investigated species or even by *de novo* sequencing approach (Figure 4). To evaluate the phosphorylation site localization, scoring algorithms are performed, including but not restricted to delta score, PhosphoRS, and Ascore, which can be integrated into different pipelines [111]. To identify glycan composition/structure, tools such as Byonic^TM^ [112], PGlyco [113], among others, can be used. Bioinformatic analysis can also assist the investigation of potential regulatory enzymes involved in specific modified sites, especially for phosphorylation (e.g., NetPhos [114], KinasePhos [115], NetworKIN [116]). Next, the identified or putative PTMs of proteins/toxins from arthropod venom can be validated or confirmed using targeted proteomics such as selected reaction monitoring (SRM) [117-119]. 

There are still a limited number of high-throughput PTM studies of arthropod venom, and even fewer studies linking this information to specific functions or structures. Hence, the following step of PTM-venomics is the biological investigation of their impact on toxicity or function activities. The integration of both approaches is referred here to as PTM-functional-venomics, and different biological approaches can be performed in this step (Figure 4). For instance, a general evaluation of *in vitro* or *in vivo* venom activity can be achieved by enzymatic removal of a specific PTM in the whole venom, preferentially combined with structural approaches to check the protein structural integrity after PTM removal. Then, fibrinogenolytic, gelatinolytic, hyaluronidase, hemolytic, platelet inhibition, enzymatic, and immunoassays can be employed to evaluate the PTM impact [68,69,120,121]. Besides, by identifying interesting modified peptides or proteins, they can be synthesized or expressed preferentially in gland venom cells to perform specific activity tests. In this step, PTM site mutation can be used to evaluate the true importance of such modification. Also, structural proteomic approaches, such as hydrogen/deuterium exchange (HDX), crosslinking, and covalent protein painting (CPP), can be used to interrogate modified protein/toxins structures and interactions [122-128]. This knowledge might also be applied to improve the application of venom proteins for biotechnological purposes. 

## Limitations in PTM-functional venomics

Although we are experiencing advances in analytical techniques used in venomics and general PTMs studies, limitations still make PTM study in toxinology research challenging. The limited amount of venom obtained from animals is one of them, especially from Arthropoda due to their small body size. To identify PTMs in venom and their impact on the toxicity and/or biological mechanism, approximately 3-5 mg of venom would be necessary, depending on the experiments. For example, around 60-100 *Loxosceles* spiders would be required to obtain this venom amount, based on the estimation that one yields 50 μg of venom proteins [129]. This number would be even more significant considering wasp and ant venoms. Therefore, after identifying modified toxins/proteins, peptide synthesis or recombinant protein expression (preferentially in gland venom cells) could assist in their functional evaluation. An alternative source for bioprospecting venom components, including PTMs, is based on organoids technology. Recently, snake venom gland organoids were generated with functionally secreted toxins [130]. We hope this technology will soon be applied to arthropods’ venom glands. 

Another significant limitation is the lack of genome sequencing of most venomous animals, which could be overcome by venom gland transcriptome. Although the number of studies and publications is increasing using this Omics approach, they are still scarce, and some data are not accessible. Besides, most of the available data is still not readily applicable for proteomics studies since their sequences are deposited without assembly and/or annotation. An alternative way to overcome the lack of a protein database is to use *de novo* sequencing, which can be employed with high-throughput algorithms such as Novor, PEAKS, and PepNovo [131]. This strategy was used in arthropod PTM-venomics [29]. However, minimum requirements are also necessary for *de novo* sequencing, such as high-resolution mass spectra [131]. 

Regarding limitations associated with PTMs, the vast majority of venomic studies focused exclusively on toxins, which discarded the identification of several PTM regulatory enzymes. We believe that the identification and characterization of such enzymes reinforce the presence of such modifications in venom toxins/proteins and would expand the current knowledge among potential modified sites. For instance, we believe that there are kinases with different specificities in arthropods to be characterized, since phosphorylation sites were identified with no predicted associated kinases, such as Tyr^111^ of Ag5 [50]. Besides, Ser, Thr, and Tyr are the most studied phosphorylated sites in Eukaryotes, but Arg may also be phosphorylated in arthropods by the action of arginine kinase that was found in several venomic studies [40,42,43]. Other amino acid residues such as His and Asp are also phosphorylated in different organisms [132,133] and future studies are necessary to verify their presence in arthropod venom proteins/toxins. 

An open question is whether the venom PTMs regulatory enzymes have specificity toward protein targets from preys and/or predators, including our proteins. In this same direction, venom components can undergo PTM within the host system as in the case of maurocalcin scorpion toxin [61], increasing the complexity to study PTM in venom. It remains unclear whether the *in vivo* host-induced PTM is a defense mechanism that reduces the toxicity/activity of venom components or eventually it improves toxin effects.

Conversely, to the identification of unpredicted sites, there are putative sites that may not occur in natural conditions. The main problem could be the site prediction-based on primary and not tertiary structure, which would lead to site inaccessibility. Therefore, it is indispensable to obtain experimental confirmation. To increase the complexity degree, the glycosylated protein may have different proteoforms with distinct effects. Since glycan structures are built with multiple carbohydrates, one carbohydrate difference can significantly change protein activity or specificity [91].

Finally, but not least, the study of PTMs in a high-throughput manner is based on specific affinity enrichment methodologies. However, only for a few PTMs an identification approach is available. Therefore, there is a long exploratory journey that we need to go through to build a comprehensive PTM-functional venomics view of arthropod venoms. 

## Conclusions

A function-to-structure approach was used at the beginning of toxinology. Omics emerged to change this scenario leading to extensive knowledge on protein/toxins present in venoms. We believe that the new step to access the venom diversity is to study PTMs that are still vastly unexplored, especially in arthropods. In our opinion, this new Omics Era should be accompanied by truly structure-to-function studies. We understand that protein annotation does not follow the rapid pace of protein identification nowadays, but a list of proteins with their PTM sites (including putative) does not add much biological information. Hence, we are still in the infancy of PTM role investigation in venom proteins. 

As we showed in this study, phosphorylation and glycosylation might have remarkable roles in venom biology, such as a neuro-immune modulatory effect. However, our knowledge about these modifications is still very scarce and further investigations are necessary to elucidate their biological meaning. Thus, with the emerging technologies discussed above, we hope that the number of combined high-throughput PTM-venomics studies associated with biological assays will increase in the future. These efforts will contribute to better understanding PTM role in arthropod venom, providing new insights on envenomation mechanisms. These findings would improve the discovery and optimization of drug-lead candidates and even find potential biomarkers for specific envenomation diagnoses. Eventually, this knowledge may lead to more precise and efficient treatment for envenomation and other diseases.

## Supplementary material

The following online material is available for this article:

Additional file 1. UniProtKB/Swiss-Prot arthropod venom proteins with posttranslational modification information. Green and yellow cells indicate experimental and putative PTMs, respectively.
